# Communicating and disseminating One Health: successes of the One Health European Joint Programme

**DOI:** 10.1099/jmm.0.001842

**Published:** 2024-07-26

**Authors:** Emma Taylor, Karin Artursson, Luca Busani, Arnaud Callegari, Jennifer Cantlay, Manuela Caniça, Elaine Campling, Dolores Gavier-Widén, Arjen van de Giessen, David Itier, Hein Imberechts, Hendrik-Jan Roest, André Jestin, Lucia de Juan, Pikka Jokelainen, Annemarie Kaesbohrer, Ann Lindberg, Alberto Mantovani, Kåre Mølbak, Wim H.M. van der Poel, Aurore C. Poirier, Ludovico P. Sepe, Stefano Morabito, Jack Whitehouse, Daniel L. Horton, Roberto La Ragione

**Affiliations:** 1 University of Surrey, School of Veterinary Medicine, Daphne Jackson Road, Guildford, UK; 2 National Veterinary Institute, Uppsala, Sweden; 3 Centre for Gender-specific Medicine - Istituto Superiore di Sanità, Viale Regina Elena 299 00161 Rome, Italy; 4 French Agency for Food, Environmental and Occupational Health & Safety, European and International Affairs Department, Maisons-Alfort, France; 5 National Reference Laboratory of Antibiotic Resistances and Healthcare Associated Infections, Department of Infectious Diseases, National Institute of Health Dr. Ricardo Jorge, Lisbon, Portugal; 6 National Institute for Public Health and the Environment, Bilthoven, The Netherlands; 7 French Ministry of Research and Higher Education, Paris, France; 8 Sciensano, The Belgian Institute for Health, Brussels, Belgium; 9 Animal Supply Chain and Animal Welfare, Ministry of Agriculture, Nature and Food Quality, The Hague, The Netherlands; 10 Department of Infectious Diseases and Immunology, Faculty of Veterinary Medicine, Utrecht University, Utrecht, Netherlands; 11 French Veterinary Academy, 34, rue Breguet, 75011 Paris, France; 12 VISAVET Health Surveillance Centre, Universidad Complutense, Madrid, Spain; 13 Animal Health Department, Facultad de Veterinaria, Universidad Complutense, Madrid, Spain; 14 Infectious Disease Preparedness, Statens Serum Institut, Copenhagen, Denmark; 15 German Federal Institute for Risk Assessment, Department Biological Safety, Berlin, Germany; 16 National Veterinary Institute, SVA, 751 89 Uppsala, Sweden; 17 Technical Advisory Group for One Health - WHO, Copenhagen, Denmark; 18 Department of Veterinary and Animal Sciences, Faculty of Health and Medical Sciences, University of Copenhagen, Frederiksberg C, Denmark; 19 Wageningen BioVeterinary Research, Department of Virology, Houtribweg 39, 8221 RA, Lelystad, Netherlands; 20 Department of Food Safety, Nutrition, and Veterinary Public Health, Istituto Superiore di Sanità, Viale Regina Elena 299 00161 Rome, Italy; 21 University of Surrey, School of Biosciences, Edward Jenner Building, Guildford, UK

**Keywords:** antimicrobial resistance, communication, emerging risks, food safety, risk analysis, zoonoses

## Abstract

The application of a One Health approach recognizes that human health, animal health, plant health and ecosystem health are intrinsically connected. Tackling complex challenges associated with foodborne zoonoses, antimicrobial resistance, and emerging threats is imperative. Therefore, the One Health European Joint Programme was established within the European Union research programme Horizon 2020. The One Health European Joint Programme activities were based on the development and harmonization of a One Health science-based framework in the European Union (EU) and involved public health, animal health and food safety institutes from almost all EU Member States, the UK and Norway, thus strengthening the cooperation between public, medical and veterinary organizations in Europe. Activities including 24 joint research projects, 6 joint integrative projects and 17 PhD projects, and a multicountry simulation exercise facilitated harmonization of laboratory methods and surveillance, and improved tools for risk assessment. The provision of sustainable solutions is integral to a One Health approach. To ensure the legacy of the work of the One Health European Joint Programme, focus was on strategic communication and dissemination of the outputs and engagement of stakeholders at the national, European and international levels.

## Data summary

https://onehealthejp.eu/

## Introduction

The application of a One Health approach recognizes that human health, animal health, plant health and ecosystem health are intrinsically connected according to the definition put forward by the One Health High-Level Expert Panel and endorsed by World Health Organization (WHO), Food and Agricultural Organization (FAO), World Organisation for Animal Health (WOAH) and UN Environment Programme [1]. This serves as the basis for expertise pertinent to public health, animal health, ecosystem health, food safety and environmental health, to come together to form a multidisciplinary and transdisciplinary collaborative approach to prevent and respond to combat complex global challenges such as foodborne zoonoses, antimicrobial resistance (AMR) and emerging threats. Tackling complex threats is imperative, and therefore, the One Health European Joint Programme (One Health EJP) was established within the European Union (EU) research programme Horizon 2020 to address these challenges, with a specific focus on AMR, foodborne zoonoses and emerging threats [2].

Consolidating existing and practical One Health approaches, developing new ones and communicating and disseminating outputs and outcomes is challenging due to the broad field of possible applications and many different audiences. In this paper, we describe a selection of communication and dissemination approaches from the One Health EJP.

Delivering results that public institutes can take up in public health, animal health and food safety across Europe was at the core of the One Health EJP approach, by developing and implementing sustainable solutions that recognize the importance and added value of collaboration. Besides building a strong One Health network of 43 multisectoral public institutions in food, veterinary and medical laboratories and institutes spanning 19 countries in Europe and the Med-Vet-Net-Association [3], training the future generation of One Health scientists was a core objective of the One Health EJP. The One Health EJP activities were based on the development and harmonization of a One Health science-based framework in the EU; therefore, the One Health EJP came to involve public health, animal health and/or food safety institutes and agencies from almost all EU Member States plus the UK and Norway. The One Health EJP focussed primarily on animal and human health, and food safety; meanwhile, as the One Health EJP started, it was still under debate on how to consider the environmental component of the One Health approach. Hence, the inclusion of environmental issues in One Health EJP activities was a work in progress and, eventually, a successful achievement: this is highlighted by the integration of environmental aspects into several joint research projects (JRPs) and other activities, including a dedicated Summer School in 2021 (see below). Many of the consortium partners have reference responsibilities and common research agendas, representing a sustainable framework for an integrated research community. Furthermore, by bringing together the major representatives of the European scientific community in the fields and those dealing with national mandates of reference and performing official research programmes in these three domains, the One Health EJP seeks to bridge the gaps in a previously fragmented research landscape. Meanwhile, the One Health EJP scientific activities developed tools, models and approaches that are transferable beyond the EU; accordingly, the One Health EJP stakeholder committee included the main relevant EU agencies [European Centre for Disease Prevention and Control (ECDC), The European Food Safety Authority (EFSA), European Medicines Agency (EMA), European Environment Agency (EEA)] and international (WHO Regional Office for Europe, FAO, WOAH) organizations.

Over the project period of 5 years and 9 months, significant progress was made by the One Health EJP partners in the development and implementation of tools. Examples include those that are surveillance focused – OHEPI-Cap [4,5]and One Health Surveillance System (OHSS) tool [6,7], genomics focused – Integrated Rapid Infectious Disease Analysis (IRIDA) [8] and communication focused [9,11] to combat nationally or globally important infectious and AMR threats now and in the future. Activities including joint integrative projects (JIPs) and JRPs facilitated harmonization of surveillance activities and laboratory methods while providing opportunities to improve accessibility and usability of tools for risk assessment and risk management. Both the JIP and JRP activities were set up in close interaction with national and European partner institutes and stakeholders, i.e. national authorities, European agencies and international bodies working across sectors. The One Health EJP established and maintained a close cooperation with these stakeholders and shared information and results from the projects covering areas of their interest. New knowledge produced by the One Health EJP supported evidence-based risk evaluation by the competent national and European authorities, informing decision-making; outcomes of the One Health EJP facilitate the operationalization of the One Health approach. The 17 sustainable development goals (SDGs) (launched by United Nations in 2015) are an urgent call for action by all low middle-income countries – in a global partnership. They recognize that ending poverty must go hand in hand with strategies that improve health and education, reduce inequality, spur economic growth and tackle climate change; the SDGs are set to be achieved by 2030. The activities of the One Health EJP are relevant to goal 2 ‘zero hunger’, 3 ‘good health and well-being’, 6 ‘clean water and sanitation’ and 17 ‘partnership for the goals’. In particular, providing evidence to support SDGs and their associated targets has been central to the development of the sustainability of the outcomes of the One Health EJP, specifically targeting 17.6 ‘*collaborate and share knowledge about science, technology, and innovations*’.

## Communications and dissemination activities

Communication and effective dissemination have been a strong thread throughout the programme and underpinned the purpose of the consortium. The creation and delivery of numerous digital publications have been an important activity to keep our news flowing and to stay connected to our many audiences [12]. For ease of use, our large most comprehensive documents – such as Annual Reports [13,15] and the Strategic Research and Innovative Agenda [16] – have been designed with clearly demarked sections and hyperlinked table of contents, signposting to project impacts. Social media offered another opportunity to communicate the activities of the consortium, with the use of Twitter (now X), LinkedIn and YouTube. An evolving communications strategy ensured that the One Health EJP outputs and outcomes were successfully disseminated for maximum uptake by the project partners and the national, EU and international stakeholders. This strategy defined the target audiences, most effective channels and platforms for internal and external communications, reporting methods and guidelines for consistent branding. More specifically, the research repository, Zenodo, was identified as the platform for uploading key deliverables and publications from each project under One Health EJP [17]. The website was an important, publicly accessible communication resource, to ensure the sustainability of the One Health EJP outcomes. It was designed to be user friendly for easy access to resources, aimed at the dissemination of scientific results (e.g. through the outcome inventory), science to policy translation outcomes, education and training activities, and One Health EJP news. Annual Scientific Meetings gathered wide scientific audiences to share and discuss the key advantages. Dissemination workshops targeted to national decision makers helped to maximize the usefulness of One Health EJP outcomes, with topics selected and based on stakeholders’ input. The One Health EJP outcome inventory (OHOI) is a publicly available database capturing all key outcomes generated under JIPs and JRPs. This repository is designed to disseminate databases covering analytical methods, host–microbe interaction, epidemiological data, risk assessment and disease control interventions [18].

### The development of a One Health EJP data management plan

The setting up of an overarching Data Management Plan (DMP) was one of the first tasks in the One Health EJP, essential in a project of this size and complexity to provide clarity and guidance. The DMP complied with the guidelines for Horizon 2020 projects on data management provided by the European Commission in the Horizon 2020 Online Manual, https://ec.europa.eu/research/participants/docs/h2020-funding-guide/cross-cutting-issues/open-access-data-management/data-management_en.htm.

All projects then set up and maintained their own DMP with an appointed leader, supported centrally. In addition, a DMP Committee was created to regularly check on the status of the project DMPs and provide advice. For the consortium’s first project call, three accessible templates were used, such as the Horizon 2020 template, the web-based tool DMPOnline.be and an Excel file specifically provided by One Health EJP.

For the second project call, there was a need to facilitate the development of project DMPs and a new online DMP software, the CDP (Collaborative Data Platform) tool (Lisam Systems, Ecaussinnes, Belgium) was introduced. The CDP tool was customized specifically for One Health EJP DMPs. This new software provided a template which improved the usability and facilitated the process to fulfil the findable, accessible, interoperable, reusable (FAIR) principles. By the end of the projects, the DMPs were uploaded to a research repository (Zenodo) for dissemination and transparency.

## The One Health EJP strategic matrix

A strategic research agenda (SRA) was developed by the consortium partners, to identify the needs of key stakeholders, and strategic links with other EU initiatives. Three domains underpin the SRA: illness and death from consumption of unsafe food (food-borne zoonoses), emergence and re-emergence of zoonotic disease in human populations (emerging threats) and the rise and transmission of resistance to existing antimicrobials in bacteria and parasites (AMR). Six themes of integrative strategic activities were then prioritized: design and implementation of surveillance activities, laboratory methods, reference material and data, interpretation of surveillance data, cross-section communication of data and action (prevention and response). JIPs and JRPs were designed to address these domains and themes.

## Joint integrative projects

To strengthen scientific capacity and the associated activities within the consortium and at the EU level, six JIPs were developed, described here under the themes they address. To avoid overlaps and identify opportunities for collaboration and alignment between these projects within the One Health EJP and with other EU initiatives, eight Cogwheel Workshops [19] were held during the first 4 years of the One Health EJP.

### Design and implementation of surveillance

#### Connecting dimensions in One Health surveillance (MATRIX)

The MATRIX project sought to create solutions to OHSSs implementation. Using a set of standardized indicators, the OH-EpiCap tool [4,5], an interactive web-based tool, helps to identify the strengths and weaknesses of epidemiological capacities of existing in-country surveillance systems and address gaps in multisectoral and multidisciplinary efforts [20,21]. The Dashboard Information Centre developed under MATRIX then provides an interactive, informative repository and manual for One Health surveillance dashboards. Similarly, the Integrate–OHSS tool also provides a step-by-step guide for creating OHSSs. These tools support the overall aim of advancing One Health surveillance by expanding on existing resources including those developed in other JIPs, identifying, and developing synergies between the sectors and enabling One Health principles to be applied.

#### One Health structure in Europe (COHESIVE)

While some European countries have successfully integrated a human and veterinary One Health structure, many other countries only did so as a reaction after experiencing a large zoonotic outbreak. In addition, the integration of the environmental component is lagging. Organizational structure and food production systems differ greatly across Europe, and it is obvious that there is no standardized blueprint for the implementation of a One Health risk analysis structure. To this end, the COHESIVE project [22] strengthened the One Health collaborative structure across risk analysis of emerging threats. One web-based tool developed is the One Health Risk Analysis System [23] to provide guidelines for setting up a One Health system for signalling, assessing and controlling zoonoses, and the implementation of this system in European countries. The expected impact is improved future integration between sectors in Europe.

### Laboratory methods

#### One Health harmonization of protocols for the detection of foodborne pathogens and AMR determinants (OH-HARMONY-CAP)

OH-HARMONY-CAP successfully delivered a strategic summary detailing the laboratory capacities for the identification of foodborne pathogens across Europe. The One Health monitoring tool was developed by OH-HARMONY-CAP to [24] achieve three main objectives. First, this tool collected information on laboratory capacity, capabilities and adaptability at both the National Reference Laboratory and primary level across Europe through the development of OHLabCap survey [25].

Second, current best practices were examined across the public health, animal health and environmental laboratories by identifying knowledge gaps and proposing ways to generate new knowledge. Lastly, the recommendations identified were communicated and disseminated to support the continued harmonization of methodologies for detection of pathogens.

### Reference material and data

#### Cross-sectoral framework for quality assurance resources for countries in the European Union (CARE)

Proficiency testing (PT) is an important component of quality assurance management of laboratories conducting diagnostics and research. The CARE project [26], developed new One Health concepts for PT of laboratories, and the quality and availability of demographic data. CARE also disseminated information about the accessibility more widely and included both reference collections and microbial collections from partners and key stakeholders [27]. To do this, the data were quality assessed and evaluated on the availability of demographic data. This then provided recommendations on how to improve data accessibility and collection to those undertaking the tasks. A specific tool was developed to identify relevant reference materials (rStainSelect).

### Interpretation of surveillance data

#### One Health suRveillance Initiative on harmOnization of data collection and interpretatioN (ORION)

ORION [28] focused on the definitions and technical coordination between sectors, specifically on surveillance outcomes and data. A framework for the harmonization of One Health descriptions was developed [29]. Through four action-driven principles (*support collaboration, knowledge exchange, data interoperability and dissemination*), this One Health Codex framework provides resources to support the adoption of the One Health principles allowing for cross-sector harmonization and interpretation of surveillance data. Other examples of tools developed include the IRIDA, an open-source end-to-end platform for public health genomics, implemented to increase collaboration and information exchange between veterinary and public health sectors in Norway. The web-based interactive OH-CRAC tool provides guidance to researchers for the type of metadata that should be collected for surveillance reports, improving the quality of reporting documentation [30]. Reach and expected impact of these integrative activities are global: The *Surveillance and Information Sharing Operational Tool* has been developed by the WOAH, FAO and WHO tripartite to support cross-sectional, multidisciplinary collaboration [31,32], with contributions from the ORION team.

#### One Health research integration on SARS-CoV-2 emergence, risk assessment and preparedness (COVRIN)

The COVRIN project was developed during the pandemic and had two main objectives: to identify the drivers of emergence and spread, and to generate data and models for risk assessment of SARS-CoV-2 [33]. The impact of COVRIN is evidenced in key outputs, which include shared molecular tests, immune assays, cell lines, and animal models. The improved sharing of research activities has resulted in optimized laboratory testing procedures and risk modelling approaches. More widely, the integration of these activities will achieve wider societal and policy impact, through improved preparedness, refined risk assessments and strategic health risk control measures. Hence, the outcomes of COVRIN contribute to building preparedness toward future possible pandemics.

### Cross-sector communication of data

#### One Health structure in Europe (COHESIVE)

Four tools have been created to support risk-based surveillance now and in the future: (i) a decision support tool for One Health risk assessment, (ii) the web-based COHESIVE information system to integrate the knowledge generated from medical and veterinary sectors and (iii) the FoodChain-Lab web application (FCL Web) for the tracing of specific food produced through the food supply chain [22]. Lastly, the COHESIVE Decision Support Tool has been designed to provide guidance to unexperienced users, by evaluating the needs of the One Health approach in question and determining the most appropriate risk assessment approach.

#### One Health suRveillance initiative on harmonization of data collection and interpretation (ORION)

Terminology and interpretation of One Health terms are a significant challenge for cross-sectional information sharing and collaboration. To address these challenges, the ORION project developed a glossary of terms; a web-based, searchable tool which conforms to the FAIR data principles [28,34].

### Action (prevention and response)

#### One Health structure in Europe (COHESIVE)

The wide variety of COHESIVE resources can be used to facilitate national and international coordination, improve the efficiency of One Health risk analysis and surveillance for zoonotic pathogens and benefit pandemic disease preparedness [22].

### OHEJP simulation exercise: a One Health scenario

Complimenting the JIPs, the One Health EJP simulation exercise (SimEx) [35,36] project is an example of using the One Health approach and tools developed in the One Health EJP in a learning exercise to address a foodborne zoonotic disease outbreak. This tabletop SimEx assessed the level of communication and collaboration between public health, animal health and food safety sectors within the 11 countries that participated in the exercise [37]. Through this exercise, OHEJP SimEx provided evidence towards the improvement of in-country One Health application to outbreak preparedness [35]. Specifically, a joint workshop entitled ‘A One Health simulation exercise as a roadmap for future foodborne outbreak preparedness’, was organized by the SimEx project team and the Science to Policy team, which was delivered to a target audience of decision makers, policymakers and risk managers to ensure the lessons learnt have future impact on preparedness in Europe.

## Joint research projects and PhDs

The majority of the scientific activity in the One Health EJP was generated through 24 JRPs and 17 PhD projects, spanning across the 3 domains of One Health EJP, foodborne zoonoses, AMR and emerging threats and the six themes developed in the SRA (Tables 1, 2 and 3). The outcomes from these research projects and PhDs have created numerous data for uptake by stakeholders, and by enabling open access to the knowledge base, data and tools. Highlights from JRPs are described according to the themes they addressed, and impact and outcomes from each PhD project are summarized in Tables1^3^.

**Table 1.** Contribution of the PhD projects under foodborne zoonoses theme

Interactive activity	PhD project	Scientific output(s) and outcome(s)	Impact and communication
Laboratory methods	**AptaTrich**	A whole-larva method which can be used to isolate aptamers specific for the muscle larvae of *Trichinella spiralis*.	Potential to enable specific and early detection of *Trichinella* in both humans and pigs.
	**TRACE**	A novel HEV probe enrichment method for the sequencing of phylogenetically different HEV strains.	Determine HEV transmission routes and prevent consumption of raw/uncooked pork products contained with HEV.
Interpretation of surveillance data	**EnvDis**	An environmental and disease model for determining the influence of weather on the incidence of salmonellosis.	Provided information on how changes in environmental, weather-related factors influence foodborne zoonoses.
	**ToxSauQMRA**	Quantitative Microbiological Risk Assessment for the detection of *Toxoplasma gondii* in pork carcases, sausages and dry ham.	An effective tool for the development of preventive strategies against *T. gondii* in the European food chain [81].
	**MACE**	Mathematical model for the surveillance of cystic echinococcosis.	Contribute to the insights and effectiveness of strategies to reduce disease prevalence, assisting evidence-based decision making.

HEV, hepatitis E virus.

**Table 2.** PhD projects and the associated research outputs and outcomes from the One Health EJP projects and related integrative strategic activities under AMR theme

Interactive activity	PhD project	Scientific output(s) and outcome(s)	Impact and communication
Laboratory methods	**LIN-RES**	Pipelines for the automatic analysis of next-generation DNA sequencing data for *Enterococci* and *Staphylococci*.	Molecular tool for the detection of AMR genes [82,83].
	**VIMOGUT**	Semi-automated *in vitro* chicken gut model.	Documented the prevalence trend and clonal spread of Extended-spectrum beta-lactamases (ESBL)-*Escherichia coli* in a broiler farm highlighting the farm environment as a potential source for ESBLs [84].
	**Codes4strains**	Innovative genomic nomenclature and typing approach, called cgLIN codes, for *Klebsiella pneumoniae*.	Contributed to epidemiological studies of diphtheria in different regions of the world, from animal and human sources [85,88].
	**Udo-FriC**	Model systems to study interactions between fluoroquinolone-resistant *Campylobacter* and chicken.	Collated UK national surveillance data and identified significant trends in fluoroquinolone resistant in *Campylobacter* strains isolated from broiler chicken.
	**KENTUCKY**	Construction of reporter plasmids and fluorescent recipient strain for the study of AMR transfer dynamics in *S*. Kentucky.	Understanding the molecular mechanisms behind the AMR genes’ transfer has the potential to improve AMR surveillance.
	**METAPRO**	A metagenomic approach for the study of the resistance determinants to next-generation aminoglycosides in ecological niches from human, animal and environmental origins.	Method of detection of the *npmA* gene (AMR) reservoirs [89].
Reference material and data	**LIN-RES**	Collection of linezolid-resistant bacteria, with their genomes and AMR profiles.	Results shared with National and European institutions (FASFC, FAMHP, AMCRA, EURL-AR, EFSA and ECDC), with the EURL-AR planning linezolid selective monitoring in EU member states.
	**VIMOGUT**	Data on the microbiota composition and diversity of broiler chicken colonised by AMR *E. coli*.	Results have been shared with the Dutch Ministry of Agriculture, Nature and Food Quality.
	**Codes4strains**	Identification and characterization of a new species of *Corynebacterium*: *C. rouxii*.	Provided bioinformatic data on the population structure of AMR in *C. diphtheriae* determining the genetic basis of resistance phenotypes.
	**HME-AMR**	Collection of AMR Enterobacterales isolated from soil, spinach and bovine milk filters samples collected from areas with high and low zinc concentrations.	Contribute to objectives of Ireland’s Second National Action Plan on Antimicrobial Resistance, and to the European Antimicrobial Resistance Surveillance Network.
	**WILBR**	Collection of pig and gull faeces from a low antimicrobial usage pig farm. Collection of *E. coli* isolated from these samples, with their AMR profiles.	Shared with the Veterinary Medicines Directorate, an executive agency of the Department of Environment, Food and Rural Affairs (Defra) within the UK Government.
	**ECO-HEN**	Collection of *E. coli* isolated from a commercial layer (egg production) farm with low antimicrobial usage and their phenotypic and genotypic AMR characterization.	Identified the predominant AMR genes.
Interpretation of surveillance data	**LIN-RES**	Data on colistin-resistant *E. coli*.	Identification of a large reservoir of *Enterococci* carrying linezolid resistance genes in food-producing animals [83].
	**UDo-FRiC**	Trend of fluoroquinolone resistance in *Campylobacter* derived from UK broiler chickens over 25 years.	The project collated UK national surveillance data from APHA and identified significant trends in fluoroquinolone resistance in *Campylobacter* strains isolated from broiler chicken.
	**ECO-HEN**	Identification and characterization of the AMR determinants and plasmids carried by the isolated *E. coli*.	Highlighted the importance of including commensal bacteria in AMR surveillance.
Cross-sector communication of data	**Codes4strains**	New bioinformatics tool: DIPHTOSCAN for the genotyping, nomenclature and toxicity prediction of *C. diphtheriae*.	Will advance the genomic epidemiology of *C. diphtheriae*, the clinical management of patients and knowledge on the links between animal and human *diphtheria* cases.

Extended-spectrum beta-lactamases (ESBL), The Federal Agency for the Safety of the Food Chain (FASFC), Federal Agency for Medicines and Health Products (FAMHP), Antimicrobial Consumption and Resistance in Animals (AMCRA)EU Reference Laboratory for Antimicrobial Resistance (EURL-AR).

**Table 3.** PhD projects and the associated research outputs and outcomes from the One Health EJP projects and related integrative strategic activities under emerging threat’s theme

Interactive activity	PhD project	Scientific output(s) and outcome(s)	Impact and communication
Design and implementation of surveillance activities	**DESIRE**	Development of an evidence-based surveillance for emerging rat-borne zoonoses in changing environments, using specific metagenomic tools in microbiome-profiling techniques.	Evidence of urban greening influence on rat-borne zoonotic disease hazard. Assessed the hazard and risk of rat-borne zoonoses for public health in the Netherlands [90].
Laboratory methods	**PEMbo**	Genomic analyses on a panel of *Mycobacterium bovis* strains in France.	Successfully collected and analysed new reference genomes of *M. bovis* strains from across France for epidemiological studies on transmission dynamics, useful for bovine tuberculosis surveillance activities.
Reference material and data	**PEMbo**	Collection and characterization of complete reference genomes of *M. bovis* strains in France.	Improved understanding of the complex biology of *M. bovis* [91].

## JRPs under foodborne zoonoses domain

### Laboratory methods

#### METASTAVA: Standardization and validation of metagenomics methods for the detection of foodborne zoonoses, AMR and emerging threats

Metagenomic analysis is commonly used to identify the potential causes of unexplained disease outbreaks. Therefore, the METASTAVA project [38] evaluated metagenomic analysis as a method to be used in public health laboratories. The project created guidelines for sequence-based metagenomic disease surveillance, proposing criteria and tools for the robust quality assurance of metagenomic work. METASTAVA also produced a range of resources for SARS-CoV-2, specifically a rapid SARS-CoV-2 whole-genome sequencing method, used during the pandemic to inform decision-making in real time at the Netherlands National Public Health Department.

#### MedVetKlebs: Klebsiella pneumoniae from ecology to source attribution and transmission control

MedVetKlebs created a novel tool, ZKIR qPCR (‘Klebsiella MALDI-TypeR’) which is capable of detecting *K. pneumoniae* in food products (e.g. chicken and vegetables) [39]. The innovative tool has made it possible to quickly detect all species of the *K. pneumoniae* complex. MedVetKlebs also created a model, which has made it possible to broadly determine foodborne or environmental source case clusters or outbreaks. MedVetKlebs project has defined the ecology of *K. pneumoniae* and has further identified the sources of infections in both animal and human hosts, to help assess possible transmission routes.

#### TOXOSOURCES: Toxoplasma gondii sources quantified

The TOXOSOURCES consortium harmonized a molecular method for detecting *T. gondii* contamination in fresh production and applied it to a large multicentre study [40]. Moreover, the potential of source-attributing serology was explored [41], microsatellite-typing was harmonized [42] and a new genotyping approach was developed to improve future assessment of risk.

### Reference material and data

#### ListAdapt: Adaptive traits of Listeria monocytogenes to its diverse ecological niches

The ListAdapt project compared genotypic and phenotypic data to identify the molecular mechanisms of adaptation of *L*. *monocytogenes*, by creating an algorithm for selecting strains to explore the diversity of strains circulating in the environment [43]. This was achieved by creating a dataset of high-quality genomes originating from strains collected in 20 European countries, to improve our understanding of *L*. *monocytogenes* ecology and diversity, with the potential to aid in surveillance at the national, European and global levels.

### Design and implementation of surveillance activities

#### AIR-SAMPLE: Air sampling, a low-cost screening tool in biosecured broiler production

Through an international multicentre collaboration, the AIR-SAMPLE project developed and validated the use of air sampling as a replacement to the commonly used sampling of faecal dropping or boot swabs for *Campylobacter* surveillance and monitoring in broiler houses [44]. The final guidelines and tool are capable of implementing air sampling and qPCR to improve detection, allowing the veterinary and food production sectors to confirm the cleanliness of broiler houses before inserting new chicks.

#### NOVA: Novel approaches for design and evaluation of cost-effective surveillance across the food chain

The NOVA project developed new surveillance tools to ensure the harmonization of existing surveillance system data use [45]. More specifically, the BfR-developed FoodChain-Lab [46] was designed to support public health institutes during foodborne zoonotic outbreak investigations, by analysing the potential contribution of suspicious food items using back- and forward-tracing in the supply chain [47]. The development of this resource offers public health institutes a route to determine the source of contamination, allowing appropriate prevention strategies to be utilized and to prevent redistribution for human consumption in Europe.

### Interpretation of surveillance data

#### ADONIS: Assessing determinants of the non-decreasing incidence of salmonella

The prevalence of *Salmonella enteritidis* is now increasing in laying hen flocks, despite a significant period of reduction [48]. While there are existing hypotheses aimed at accounting for this increase, the ADONIS project identified the possible causes of the increase in *S. enteritidis*. The project created a multicriteria decision analysis model to assist with decision-making by ranking potential determinants and options for the intervention for the *S. enteritidis* trend in Europe according to relative importance.

#### BeONE: Building integrative tools for One Health surveillance

The BeONE project developed integrated One Health solutions which allow for molecular and epidemiological data to be analysed, visualized and interpreted correctly by experts from multiple disciplines and sectors. BeONE datasets were created for zoonotic pathogens (*L*. *monocytogenes, Salmonella enterica, Escherichia coli* and *Campylobacter jejuni*), to potentiate the genetic diversity necessary for certain genomic analysis, representing a useful asset in future surveillance and research-oriented studies [49]. Additionally, the tool ReporTree facilitates and accelerates the production of surveillance-oriented reports, which contributes to a sustainable and efficient public health genomics-informed pathogen surveillance systems.

#### DiSCoVeR: Discovering the sources of Salmonella, Campylobacter, verocytotoxigenic E. coli and AMR

Experts from the microbiology, bioinformatics and epidemiology sectors, across a range of disciplines, worked together on the DiSCoVeR project to identify and map knowledge gaps and address the challenges associated with source attribution [50]. These unique multicountry datasets are open access and provide researchers with novel insights into the epidemiology of foodborne hazards, which may further inform on strategies to deal with future outbreaks in Europe.

### TOXOSOURCES: Toxoplasma gondii sources quantified

Using a multidisciplinary approach, coupled with novel and improved methods and extensive data collection efforts, TOXOSOURCES investigated different sources of *T. gondii* infections [40]. The project developed and applied a quantitative microbiological risk assessment model, which can provide quantitative estimates of the contribution of different sources and transmission routes of *T. gondii* infections.

#### MedVetKlebs: Klebsiella pneumoniae from ecology to source attribution and transmission control

An additional output of MedVetKlebs was a multicentre study that produced a novel isolation strategy for *K. pneumoniae* [39]. The study identified that the presence of *K. pneumoniae* in salad and chicken meat was high across the food sector. These findings need to be further explored to define possible control strategies and to understand the degree at which food contamination by *K. pneumoniae* contributes to human infection.

### Cross-sector communication of data

#### BIOPIGEE: Biosecurity practices for pig farming across Europe

The BIOPIGEE project identified effective biosecurity methods to control against *Salmonella* and hepatitis E virus (HEV) in European pig farms and abattoirs [51]. Comprehensive literature reviews, field and experimental studies, expert opinion surveys and risk modelling were used. Workshops and questionnaires were conducted, and two interactive biosecurity check lists were developed and implemented. The materials should provide reliable and cost-efficient guidance on the reduction of *Salmonella* and HEV spread in animals and humans, information for monitoring and regulation to advance One Health.

#### NOVA: Novel approaches to design and evaluation of cost-effective surveillance across the food chain

NOVA produced a mathematical model capable of simulating sampling schedules and standard laboratory procedures to inform on the time required to detect emerging infections [45]. Furthermore, the model took in a range of factors, including the location of contamination and consumer food purchase data.

#### Action (prevention and response)

#### MoMIR-PPC: Monitoring the gut microbiota and immune response to predict, prevent and control zoonoses in humans and livestock in order to minimize the use of antimicrobials

The MoMIR-PPC project provided a cost-effectiveness evaluation for interventions such as pre- and probiotics to prevent and control zoonoses with the goal of minimizing antimicrobial use [52]. MoMIR-PPC identified the role of animal-animal recontamination and evidenced that infection was, in part, driven by host factors and the gut microbiota. A mathematical model of indirect transmission of bacteria between broilers was developed, assessing the bio-security-based intervention strategies against *Campylobacter* and *Salmonella*. Additionally, this project created several preventive measures based on the use of pre- and probiotics to prevent salmonellosis, as an alternative to antibiotics. The outcomes of MoMIR-PPC may improve EU industry sustainability and safe trade by providing information and tools leading to on-farm control of *Salmonella*.

#### BIOPIGEE: Biosecurity practices for pig farming across Europe

A BIOPIGEE Information Materials for Farming Schools was developed to evidence good biosecurity practices on pig farms in Europe, accompanied with pictures and research findings to support the effectiveness of these strategies through a BIOPIGEE Glossary, BIOPIGEE Biosecurity Protocol, BIOPIGEE Checklists, BIOPIGEE Slaughterhouse Guidance Manual and the BIOPIGEE Cost-effectiveness Support Tool [51]. These resources were created with users in mind, ensuring that these tools could be used by diverse categories, such as farmers associations and individual farmers, veterinary practitioners, researchers, policymakers and industry stakeholders, thus improving the general knowledge of biosecurity by breaking down knowledge barriers.

## JRPs under the AMR domain

### Laboratory methods

#### IMPART: Improving phenotypic AMR testing by development of sensitive screening assays for emerging resistances and setting missing epidemiological cut-off values (ECOFFs)

Complementing the European Commission’s Action Plan against the ongoing threat of AMR, the IMPART project worked across four topics which supported the development of phenotypic methods for the detection of AMR [53]. The project also updated and improved detection protocols for AMR-producing Enterobacterales. In close cooperation with the European Committee on Antimicrobial Susceptibility Testing (EUCAST), new epidemiological cut-off values (ECOFFs) were established for *Staphylococcus pseudintermedius*, *Staphylococcus hyicus, Mannheimia haemolytica* and *Pasteurella multocida*, and are now available on the EUCAST website [54].

#### FARMED: Fast AMR and mobile-element detection using metagenomics for animal and human on-site tests

The FARMED project used the MinION technology (Oxford Nanopore Technologies) to address the limitations of short-read technology which cannot reliably identify individual genes to a specific organism [55]. By using the MinION technology, the project was able to identify a multitude of bacterial species and a range of AMR genes. Therefore, by using long-read metagenomics, FARMED outputs expected to have impact through a better detection and characterization of AMR, pathogens and genetically modified microorganisms [56].

#### WORLDCOM: Development of new tools for real-time detection of zoonotic bacteria and AMR in veterinary, human and environmental sources

The WORLDCOM project developed One Health-based technology that combined mobile technology with diagnostic tools to allow for rapid on-site AMR detection in both animal and human populations, and the environment [57]. Hence, WORLDCOM deliverables include a portable nucleic acid diagnostics workstation and assays for the detection of AMR biomarkers for on-site AMR detection.

#### FULL-FORCE: Full-length sequencing for an enhanced effort to map and understand drivers and reservoirs of AMR

The FULL-FORCE project supplied hands-on training to EU partners, to perform plasmid sequencing, in an acknowledgement to the shift away from microbiological surveillance to genomic DNA-based surveillance of infectious diseases [58]. The deliverables and outcomes included laboratory protocols for the creation of long-read sequencing dataset.

#### FED-AMR: The role of free extracellular DNA in dissemination of AMR over ecosystem boundaries along the food/feed chain

The role of free extracellular DNA is potentially crucial but under researched [59]. FED-AMR developed harmonized protocols for *C. difficile* collection and isolation in animal, food and environmental samples to assess the development of AMR.

### Reference material and data

#### ARDIG: Antibiotic resistance dynamics: the influence of geographic origin and management systems on resistance gene flows within humans, animals and the environment

The use of antimicrobial agents and associated AMR prevalence can differ greatly from country to country. The ARDIG project assessed these differences across the humans, animals and the associated environment (poultry and pig slaughterhouses, pig farms) in six countries and identified potential routes for transmission of resistance [60]. This resulted in a large collection of genomes that can be used as reference material for AMR confirmation.

#### WORLDCOM: Development of new tools for real-time detection of zoonotic bacteria and AMR in veterinary, human and environmental sources

In addition to a diagnostic platform, the project generated a reference database of sequences from currently circulating AMR pathogenic strains [57].

### Interpretation of surveillance data

#### ARDIG: Antibiotic resistance dynamics: the influence of geographic origin and management systems on resistance gene flows within humans, animals and the environment

The collection of genomic reference data resulted in a comparison between antimicrobial usage (AMU) and AMR data to improve AMR surveillance [60]. The results suggest that measures put in place to combat AMR, including the reduction in AMU in each country, have been effective. A workshop held with experts in the field, allowed recommendations from ARDIG, to improve ‘One Health’ surveillance strategies.

#### FULL-FORCE: Full-length sequencing for an enhanced EFFORT to map and understand drivers and reservoirs of AMR

The FULL-FORCE project generated an open-source bioinformatics pipeline for automated analyses of sequencing data and plasmid assembly: the Full Force Plasmid Assembler [58]. Outcomes are data on plasmid structure and variability of drug-resistant organisms, and a toolbox for single-molecule real-time sequencing for AMR surveillance, including protocols and a bioinformatics pipeline.

#### RaDAR: Risk and disease burden of AMR

There is a need to integrate data on AMR into consensus estimates for source attribution, risk analysis and disease burden estimations. Therefore, the RaDAR project generated such consensus estimates via the integration of data, which was available from cross-sectional sources. As an output, the project developed COMPASS, a large database of AMR plasmids from a range of different bacterial species and sources [61].

### Cross-sector communication of data

#### FED-AMR: The role of free extracellular DNA in dissemination of AMR over ecosystem boundaries along the food/feed chain

The FED-AMR generated new data on the transmission of AMR in an agricultural setting and completed a systematic review of the factors which influence the prevalence of AMR in the environment [59,62]. These tools are now used cross-sector by consortium partners and been disseminated externally through scientific publications and knowledge exchange activities.

#### RaDAR: Risk and disease burden of AMR

The COMPASS database will help researchers from all sectors understand the genetic plasticity and transmission routes of plasmids, which are crucial in the fight against the spread of AMR pathogens [61].

## JRPs under the emerging threats domain

### Laboratory methods

#### TOX-detect: Development and harmonization of innovative methods for comprehensive analysis of foodborne toxigenic bacteria, i.e. Staphylococci, Bacillus cereus and Clostridium perfringens

The sectors of human, animal and environmental health, and food safety were included in the TOX-detect project, allowing for a One Health approach [63]. The result of this project was the development and harmonization of methods for detecting toxin expression and production by toxigenic bacteria across all One Health pillars to improve surveillance.

#### MAD-Vir: Metagenomic array detection of emerging virus in EU

The MAD-Vir project developed metagenomics array technology, providing rapid identification of all known viruses, and associated viral families, as a cheaper alternative to NGS that is applicable to a clinical setting [64]. Early identification of zoonotic pathogens is enabled via the development of a microarray chip allowing new virus probes to be updated. This is currently implemented in four European laboratories.

#### TELE-VIR: Point-of-incidence toolbox for emerging virus threats

The TELE-Vir project developed the point-of-evidence toolbox that allows for the rapid identification of new and known viral threats occurring in animals and humans, notably applying methods that can be used in the field or in a geographical area where laboratory infrastructure is poor [65]. This facilitates immediate responses by decision makers when dealing with a potential or emerging outbreak, supporting overall outbreak preparedness across Europe.

#### IDEMBRU: Identification of emerging Brucella species: new threats for human and animals

An extensive evaluation and further understanding of the emergence of non-classical *Brucella* and the associate reservoirs are needed to continue protecting people and animals from the disease. The IDEMBRU project developed a toolbox that generates data from emerging *Brucella* outbreaks to be used as a resource guide for the characterization of the pathogen [66,67]. Guidance includes optimized methods for molecular typing, protocols for whole-genome sequencing and data analysis tools. Developing and implementing cost-effective interventions that are sustainable across a range of settings are needed to combat global emerging threats.

#### MEmE: Multi-centre study on Echinococcus multilocularis and Echinococcus granulosus s.l. in Europe: development and harmonization of diagnostic methods in the food chain

Functioning as a multicentred collaborative project, MEmE produced standard operating procedures for the sampling of experimentally and naturally infected hosts, both definitive hosts and intermediate hosts, validated multiple parasitological molecular diagnostics tools and completed comparative studies of new molecular tools [68]. The MEmE project also provided an update on the burden of neglected pandemics of cystic and alveolar echinococcosis, further drawing attention to the need for a cross-discipline, collaborative approach to disease control and elimination [69].

#### PARADISE: Parasite detection, isolation and evaluation

Foodborne parasites such as the protozoa *Cryptosporidium* and *Giardia* are attributed to major outbreaks of gastrointestinal disease in Europe. The PARADISE project successfully provided improved genotyping schemes including multilocus sequencing typing and whole-genome sequencing data for *Cryptosporidium parvum* and *Giardia duodenalis* to inform outbreak investigation and source attribution [70].

#### SUSTAIN: Scientific understanding of the policy process for transboundary integration and institutionalization of the One Health across EU Member States

An additional PhD, designed to complement the three domains was cross-disciplinary in nature (social science and public health) [71]. This PhD project aimed to understand drivers and constraints for institutionalization of the One Health approach across Europe. The results of this project have identified where knowledge exchange between sectors (public health, veterinary health and environment) can improve operationalization, create collaborative thinking and support One Health approaches.

## Science to policy translation and targeted communication to key stakeholders

A key purpose of the activities generated within the One Health EJP consortium was to provide scientific evidence to risk assessors, risk managers and policymakers and to communicate the scientific evidence to all those working within the field of One Health and the wider society. New policy development requires support to integrate One Health and ensure that international collaboration is accounted for, to truly ensure that science to policy transition occurs. The One Health EJP had a specific team working on science-to-policy translation, keeping regular interactions with the stakeholders, with particular focus on EU agencies (ECDC, EFSA, EEA, EMA) and international organizations (FAO, WOAH, WHO Regional Office for Europe).

Platforms established for interaction with the stakeholders, importantly the half-yearly Stakeholders Committee meetings, were a platform for exchange among the stakeholders, a prelude to institutional initiatives. For example, the bridges built with EU agencies contributed to the creation of One Health cross-agency task force [72]. Research activities of the consortium were set up to address stakeholders’ knowledge gaps and needs, and added value of the developed solutions could be demonstrated thanks to solid interactions at science–policy interfaces. The One Health EJP Communications Team supported science to policy translation via communication-based events, dissemination activities and workshops, promotional social media campaigns, newsletter articles, flyers and video conference links. In the final year of One Health EJP, a large conference ‘Collaborating to face future One Health challenges in Europe’ was organized to facilitate high-level discussions with stakeholders on a variety of One Health topics at science–policy interfaces [73].

## Education and training activities

The education and training activities were aimed at early career researchers, classified as less than 5 years post-PhD completion, professional researchers and students from Bachelor to PhD level. The goal of these activities was to provide a sustainable framework for the integration of education in research groups and reference laboratories across the consortium network. This was achieved following two paths: (i) improving and disseminating proficiency on the core fields and activities of the One Health EJP and (ii) broadening the One Health vision beyond the core One Health EJP topics. Another aspect was achieving a more global view of One Health, going also beyond the EU boundaries; accordingly, the activities involved teachers and students from outside the EU, including low middle-income countries. Thus, the activities aimed at providing a platform for the next generation of One Health students and researchers.

### One Health EJP short-term missions

The One Health EJP short term missions (STMs) were co-funded travel grants which facilitated the exchange of scientific expertise, methodologies, equipment and facilities. The aim of these missions was to harmonize the existing approaches and methodologies within the One Health EJP. These STMs drove research forward in a collaborative and non-duplicative fashion to strengthen the scientific capacity within the One Health EJP and to also contribute to the future prevention, preparedness, detection and response of the EU to foodborne and other emerging threats across human–animal–environmental sectors.

The One Health EJP co-funded a total of 20 STMs, which involved 23 researchers and a total of 28 institutes within the One Health EJP consortium. For each STM, a case study, summarizing the work and its outcomes, was published [74,77].

### One Health EJP summer schools

A flagship component of the education and training activities were yearly One Health EJP summer schools. These schools, in addition to education and training, provided networking opportunities for students, PhD researchers and early careers researchers working within a veterinary, human medicine, or biological science, social sciences, public health and economic sectors to meet peers and researchers from across disciplines and settings. The purpose was to deliver knowledge, and advance skills, centred around solutions for global challenges to infectious disease in human, animal and environment, with the focus on major One Health themes: One Health risk assessment and operationalization (2019, on-site event in UK); global health (2020, online event); the environmental pillar (2021, online event) and sustainability in One Health (2022, online event, UK). The 2021 summer school organized by the One Health EJP partner Instituto Superiore di Sanità (Italy) was the first training event specifically dedicated to the environmental ‘pillar’, a critical underdeveloped component of the One Health framework [78].

### One Health EJP doctoral programme

The One Health EJP Doctoral Programme has supported 17 PhD projects, selected from two funding calls. The doctoral programme enabled PhD researchers to meet international and multidisciplinary collaborators, creating their own independent network of support. This was achieved through interaction with the JIPs and JRPs, allowing for peer–peer learning, opportunities to share knowledge and expertise from professionals across the consortium. In total, 34 publications have been generated from the PhD projects to date with more anticipated.

### One Health EJP and continuing professional development

Continuing professional development (CPD) is the process designed to record and reflect upon the skills, knowledge and experience gained during work. The One Health EJP’s CPD modules covered several themes in One Health. The CPD opportunities primarily targeted topics relevant to the effective implementation of One Health actions, which were ‘outbreak preparedness’, ‘rapid diagnostics and harmonization of diagnostic tests’, ‘big data’ and ‘digital innovation’.

## Discussion: future legacy of the One Health EJP activities

The provision of sustainable solutions is integral to a One Health approach. Throughout the years, an overarching objective of the One Health EJP was to ensure that the scientific outputs, databases and tools developed, in a cross-sectoral way, remain sustainable beyond the project lifetime and are taken up by stakeholders. Therefore, to ensure the legacy of One Health EJP work, the strategy was to communicate these outputs and encourage stakeholders at the national, European and international levels to engage. To achieve this objective, the consortium worked closely with important EU stakeholders both through the formal stakeholders committee and beyond. The fruitfulness of the interaction with the stakeholders is demonstrated, e.g. by the exchanges at the One Health EJP conference ‘Collaborating to face future One Health challenges in Europe’, where current status of European One Health, the role of the One Health EJP and the policy directions to address the challenges of tomorrow were discussed [73].

To maximize the future impact of the One Health EJP outcomes, these are continuously disseminated through the project’s stakeholders and to scientific communities to ensure continued implementation. In addition, the support and involvement of WHO, WOAH and FAO are instrumental to sustainability by providing a global outreach to activities and outcomes. More recently, the bilateral meetings were set up with contacts of key stakeholders. In these meetings, stakeholders were encouraged to identify profitable One Health EJP outcomes, liaise with the Med-Vet-Net Association and consider presenting outcomes described in the OHOI as examples of good One Health practice. There are also several key initiatives and projects under the One Health EJP that specifically allow for the continuation of the One Health collaboration network after the end of the programme in 2023. The Med-Vet-Net Association, a partner in the consortium, will act as a One Health knowledge exchange forum to preserve and consolidate the network.

Multiple European Partnerships (EUPs) are also excellent opportunities for ensuring the sustainability of the outcomes and impact of the One Health EJP, with the specific focus on Horizon Europe and contributing significantly to achieving the EU’s political priorities. Several partnerships (especially partnerships on Animal Health and Welfare, Sustainable Food Systems, Pandemic Preparedness, One Health AMR and Biodiversa+) highly relevant to the topics of the One Health EJP and many of the consortium outcomes are expected to be taken up by the EUPs, ensuring the legacy of those activities remains. Rather than the focus of a single, however important initiative, this will make One Health, in a broad-range approach to many different fields, as endorsed by EFSA and the other EU agencies [72].

The Communications Team created a strong, recognizable brand with a global reach that contributes to the impact and sustainability of the One Health EJP outcomes beyond the end of the programme. Targeted dissemination activities to One Health networks through multiple digital and physical methods have been fundamental to sharing our outcomes to relevant audiences and have generated a global interest in the consortium.

The new and strengthened One Health capacities and capabilities at the national and European levels have improved preparedness and paved the way for multidisciplinary approaches to complex challenges. The efficiently disseminated outcomes of One Health EJP will continue to contribute to the One Health field in Europe and globally. The connections and networks built during this programme will be the basis of numerous future collaborations and communications.

The One Health EJP consortium guides others towards continued initiatives using a One Health approach and encourages further uptake of the instruments and opportunities arising from the outcomes. For example, the OH-EpiCap tool [4,5] from JIP MATRIX is being applied in a EU4Health-co-funded project OH4Surveillance to evaluate surveillance systems in several countries [79].

The JRPs, JIPs, PhD projects and their associated outcomes have highlighted the importance of creating trust among public institutions, especially when cross-border outbreaks are managed, and will ensure consistency in the application of techniques across European laboratories and stakeholders. Furthermore, the overall success of the One Health EJP has been achieved through extensive, multidisciplinary collaborations among public health, animal health and food safety sectors; consistent engagement is an essential component of the One Health approach. This is evident in the many references to One Health EJP projects at One Health symposia [80]. However, identifying the stakeholders and wider collaborators at the beginning of a project is key for successful and sustainable intersectoral collaboration. While an active and intersectoral surveillance system may already exist in some settings, it is imperative to ensure that those individuals involved are using standardized guidelines, methods and consistent terminology, and are in receipt of training. In addition, harmonized datasets allow for cross-sector data sharing and lessons to be learnt. Lastly, it is recognized that developing capability, building capacity and reinforcing preparedness should be completed during ‘peace time‘, thus creating trust among institutions and between sectors.

## Abbreviations

AMR: antimicrobial resistance
AMU: antimicrobial usage
CDP: Collaborative Data Platform
CPD: continuing professional development
DMP: Data Management Plan
ECDC: European Centre for Disease Prevention and Control
ECOFF: epidemiological cut-off value
EEA: European Environment Agency
EFSA: European Food Safety Authority
EMA: European Medicines Agency
EU: European Union
EUCAST: European Committee on Antimicrobial Susceptibility Testing
EUP: European Partnership
FAIR: findable, accessible, interoperable, reusable
FAO: Food and Agricultural Organization
FCL Web: Food Chain-Lab web application
HEV: hepatitis E virus
IRIDA: Integrated Rapid Infectious Disease Analysis
JIP: joint integrative project
JRP: joint research project
OHOI: One Health EJP outcome inventory
OHSS: One Health Surveillance System
One Health EJP: One Health European Joint Programme
PT: proficiency testing
SDG: sustainable development goal
SimEx: simulation exercise
SRA: strategic research agenda
STM: short term mission
WHO: World Health Organization
WOAH: World Organisation for Animal Health
